# Comparison of Low-Cost 5G Electromagnetic Field Sensors

**DOI:** 10.3390/s23063312

**Published:** 2023-03-21

**Authors:** Kenneth Deprez, Loek Colussi, Erdal Korkmaz, Sam Aerts, Derek Land, Stephan Littel, Leen Verloock, David Plets, Wout Joseph, John Bolte

**Affiliations:** 1 Department of Information Technology, IMEC-WAVES, Ghent University, 9052 Ghent, Belgium; sam.aerts@ugent.be (S.A.); leen.verloock@ugent.be (L.V.); david.plets@ugent.be (D.P.); wout.joseph@ugent.be (W.J.); 2 Radiocommunications Agency, 9726 AH Groningen, The Netherlands; loek.colussi@agentschaptelecom.nl; 3 Research Group Smart Sensor Systems, The Hague University of Applied Sciences, 2521 EN Den Haag, The Netherlands; e.korkmaz@hhs.nl (E.K.); d.d.land@hhs.nl (D.L.); s.j.littel@student.hhs.nl (S.L.); j.f.b.bolte@hhs.nl (J.B.); 4 National Institute for Public Health and the Environment (RIVM), 3721 MA Bilthoven, The Netherlands

**Keywords:** 5G new radio, electromagnetic fields, exposure assessment, measurement equipment, RF measurement, RF-EMF

## Abstract

This paper compares different low-cost sensors that can measure (5G) RF-EMF exposure. The sensors are either commercially available (off-the-shelf Software Defined Radio (SDR) Adalm Pluto) or constructed by a research institution (i.e., imec-WAVES, Ghent University and Smart Sensor Systems research group (S³R), The Hague University of Applied Sciences). Both in-lab (GTEM cell) and in-situ measurements have been performed for this comparison. The in-lab measurements tested the linearity and sensitivity, which can then be used to calibrate the sensors. The in-situ testing confirmed that the low-cost hardware sensors and SDR can be used to assess the RF-EMF radiation. The variability between the sensors was 1.78 dB on average, with a maximum deviation of 5.26 dB. Values between 0.09 V/m and 2.44 V/m were obtained at a distance of about 50 m from the base station. These devices can be used to provide the general public and governments with temporal and spatial 5G electromagnetic field values.

## 1. Introduction

Wireless communication infrastructure is changing with the advent of the newest wireless telecommunication network, 5G New Radio (5G NR). 5G NR is currently either being rolled out or on the verge of installment. Public uncertainty and anxiety about RF-EMF exposure are growing due to the communication about 5G NR. To overcome growing concerns in the rollout of RF-EMF infrastructure, RF-EMF exposure must be accurately monitored both spatially and temporally.

Due to the advent of the Internet of Things (IoT) and the increasing number of people using telecommunication technologies, the number of connections has increased over the last few years, facilitating the need for a higher number of devices that can be serviced per base station (BS) with a higher throughput per connection [1]. Due to the increasing number of connections, the number of BSs also increased [2], adding to the growing uncertainty and anxiety.

Cities are currently exploring smart sensor network deployments to monitor their daily exposure (e.g., sound, air quality, temperature) with low-cost, low-complexity sensors. These geographically distributed sensors rely on radio frequency (RF) electromagnetic fields (EMF) to ensure wireless connection and connection to the IoT [3,4].

Governments can rely on geographically distributed sensors to measure RF-EMF exposure over a large period of time at various locations. In recent years, RF-EMF exposure monitoring networks have been set up in European countries such as France, Spain, Greece and Belgium [5].

Aerts et al. [6] analyzed the output of the low-complexity sensors that were installed in the city of Antwerp, Belgium. The high variability of the measured exposure values observed over time further underlined the need for temporal monitoring. However, the installed sensors were not able to measure the new frequencies that 5G NR introduced (e.g., frequency band n78, 3.3 GHz–3.8 GHz). Consequently, new sensors have been developed that can measure at these higher frequencies and can be used to inform both public and government bodies of the new 5G RF-EMF exposure. This paper will compare different low-cost sensors that can measure this FR1 5G exposure. The sensors are either commercially available (off-the-shelf Software Defined radio (SDR) Adalm Pluto) or constructed by a research institution (i.e., imec-WAVES, Ghent University and Smart Sensor Systems research group (S³R), The Hague University of Applied Sciences). These sensors are part of European projects NEXTGEM and GOLIAT. The goal of this study is to compare design choices (LOG detector vs RMS detector vs SDR) and evaluate the performance. These results can then be used within these projects to set up RF-EMF monitoring networks. A comparison has been performed in both a Gigahertz Transverse Electro Magnetic (GTEM) cell as well as in-situ near a 5G NR base station (Green Village in Delft).

## 2. Materials and Methods

The goal of the in-lab testing was to determine the calibration factors of the different sensors and investigate how the sensors reacted to a known emitted field in the GTEM cell. The goal of the in-situ testing was to evaluate how the sensors perform in a real-life network, in the presence of known control signals and with induced traffic on nearby user equipment (UE), i.e., a 5G-enabled phone.

### 2.1. Measurement Equipment

The investigated sensors are described below. An SDR is a radio communication system in which the behavior relies on software instead of implemented hardware (e.g., filters, amplifiers, detectors). The other sensors are hardware-reliant sensors that can each capture up to four frequencies. The advantages and disadvantages of both systems are discussed in the discussed in Section 3.3 on the sensors.

#### 2.1.1. SDR Sensor

**Figure 1:**
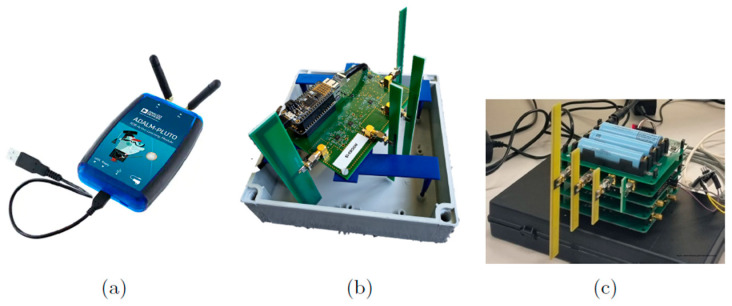
(**a**) Adalm Pluto SDR; (**b**) WAVES sensor; (**c**) S^3^R sensor.

The SDR sensor consists of an Adalm Pluto (Figure 1a) easy-to-use active learning module that can help in understanding the fundamentals of SDR, RF, and wireless communications. The SDR sensor works as a portable lab, which is small enough to fit in the pocket of one’s shirt and can be controlled from, e.g., a Matlab or Python environment. The SDR sensor can cover RF analog signals from 325 MHz to 3800 MHz at up to 61.44 Mega Samples per second (MSPS) with a bandwidth of up to 40 MHz. The Adalm Pluto is equipped with an AD9363 chip, which has the aforementioned frequency range. However, it is possible to programmatically “trick” the Adalm Pluto to think it has the AD9364 installed, which has a frequency range of 70 MHz to 6 GHz. Hence, this new frequency range can be measured with the original chipset. The Adalm Pluto can be used to both send and receive data, has an analog to digital conversion resolution of 12 bits and a detection limit of −98 dBm and has a dynamic range of >80 dB. The Adalm Pluto is combined with two types of receiving antennas: JCG401 (828–984 MHz and 1710–2170 MHz) and W5150 (617–6000 MHz). In this study, these are indicated as SDR1 (JCG401) and SDR2 (W5150).

#### 2.1.2. WAVES Sensor

Deprez et al. [7] published an overview of the single-axis WAVES sensor (Figure 1b). Here, only the main hardware is discussed. The sensor uses four half-wavelength dipole antennas with a frequency-specific Surface Acoustic Wave (SAW) filter. The output of the filter is then fed to a true-root-mean-square (tRMS) RF detector, which has a dynamic range of at least 60 dB for the four frequency bands. The lower detection levels are unique per band and vary between −65 dBm and −56 dBm. A 12-bit ADC is used which has a sample speed of 3.3 kSPS. The frequency bands that are measured in the investigated sensor are the following: 791–821 MHz, 925–960 MHz, 1805–1880 MHz, 3550–3700 MHz. Two identical WAVES sensors are considered in this study (i.e., WAVES1 and WAVES2).

#### 2.1.3. Smart Sensor Systems Research (S³R) Group Sensor

The sensor of the Smart Sensor Systems research (S³R) group also relies on hardware configurations (Figure 1c) [8]. Here, each function (e.g., power, RF detection, processing) has a dedicated PCB design. This sensor thus consists of three stacked PCBs, which leads to a modular sensor design where a change in one of the functions can be made without interference to the whole sensor. The RF detectors are logarithmic power detectors with a dynamic range varying between 65 and 70 dB for the four frequency bands and the lower detection levels vary between –60 dBm and –65 dBm. For the processing of the data, ESP32-S2 is used as a microcontroller which has a built-in 13-bit ADC. The signals are internally sampled at a sampling speed of 1000 Hz and averaged over 1 s to store the data. The S³R sensor is designed to measure four 5G downlink frequency bands for the Netherlands: 758–788 MHz, 1452–1492 MHz, 2110–2170 MHz, and 3500–3700 MHz (not yet in service). The sensor node is equipped with a battery to be able to operate for mobile measurement purposes, has a WiFi connection to read the real-time measurements on a mobile device (e.g., laptop, tablet, phone) and also to upload the measurement results to a server. Furthermore, it has an SD card to save the measured data. As receiving antennas, four half-wave dipole PCB antennas are designed.

In the remainder of the paper, we will denote the different RF-EMF sensors as SDR, WAVES, and S³R sensors.

### 2.2. Benchmark Measurement Equipment

Two measurement setups have been used to validate the performance of the sensors mentioned in the previous chapter. These will be the baseline for further analysis. Both are commercially available setups and have been used in a multitude of studies [9,10,11].

#### 2.2.1. Spectrum Analyzer with Tri-Axial Antenna

A Rohde & Schwarz FSV spectrum and signal analyzer FSV30, with option R&S FSV-K14 ‘spectrogram mode’ was connected to a Clampco Sistemi AT6000 tri-axial antenna. This setup has a frequency range of 400 MHz to 6 GHz. In order to capture the total electric-field level, the three orthogonal components (X, Y and Z) of the electric field must be measured. The Clampco antenna can internally switch the respective axis, which allows for sequential measurement of the components. The settings for the SA setup can be found in [11].

#### 2.2.2. Field Strength Analyzer

The Narda SRM3006 is a portable narrowband field strength analyzer. It is a complete and easy-to-use omnidirectional test system consisting of a base unit and measuring antennas. It is the most widely used measurement device by governments to assess EMF exposure. For this study, a tri-axial antenna (3502/01) with a frequency range of 420 MHz to 6 GHz was used. However, for in-depth analysis of the 5G NR signal, compared to the FSV-30, the parameters are less adjustable [11].

### 2.3. Gigahertz Transverse Electro Magnetic (GTEM) Cell

**Figure 2:**
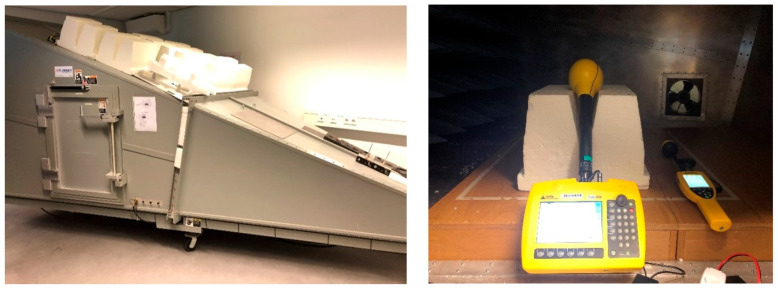
GTEM cell outside and inside. Benchmark equipment (SRM3006) inserted in the GTEM cell.

The GTEM cell was used to determine or verify the calibration factor of the 5G sensors under test. Furthermore, sensitivity, repeatability, linearity and accuracy were investigated. The GTEM cell (Figure 2) was connected to a signal generator. The GTEM generates a vertically polarized TEM wave, corresponding to the direction of the antennas on the devices under test (DUTs). The technical characteristics of the used GTEM can be found in [12]. The DUTs were placed inside the GTEM cell as shown in Figure 2 and described in [12,13].

Three scenarios were investigated using the GTEM cell. The SRM3006 was used as a baseline for the GTEM cell measurements, which means that it was also placed inside the GTEM cell at the same position as the DUTs for all the scenarios. The SRM3006 captured the full bandwidth of the signal generated in the GTEM cell by means of an RMS detector.

#### 2.3.1. Downlink Power Sweep

As the hardware sensors can measure multiple frequency bands, eight frequencies were tested sequentially. These frequencies were chosen to correspond to the center frequencies of the frequency bands used by the Mobile Network Operators (MNOs) in the Netherlands and Belgium: 773 MHz; 806 MHz; 940 MHz; 1472 MHz; 1840 MHz; 2140 MHz; 2655 MHz; 3600 MHz. The signal source consists of an R&S SGMA vector signal generator that provides a 10 MHz wide Additive white Gaussian noise (AWGN) signal to mimic the characteristics of real telecom signals. Hence, a frequency sweep was performed that allows in-band and out-of-band downlink exposure measurements by the hardware sensors. The SDRs are able to measure all the frequencies within their range. During all measurements, a field of approximately 1 V/m was present at the measurement position inside the GTEM, to either determine or verify the calibration factor of the sensor (SDR sensor, S³R sensor, and WAVES sensor). For every frequency, the field strength was monitored by a field strength analyzer.

In addition to the frequency sweep, a power sweep was performed for each frequency. Five field strengths have been tested to determine the linearity and responsivity of the DUT. The field strengths that were used are: 1 V/m; 0.3 V/m; 0.1 V/m; 0.03 V/m and 0.01 V/m.

#### 2.3.2. Uplink Frequency Sweep

All but two (i.e., 1472 and 3600 MHz) of the considered frequencies use frequency division duplex (FDD). FDD entails that the uplink is sent in a different frequency band than the downlink. Hence, the corresponding uplink frequencies were also swept. It is important to remark that the sensors (both hardware sensors (WAVES and S³R) and SDR) focus on downlink (i.e., base station) exposure, so these uplink tests should lead to no response in these sensors. The considered uplink frequencies, corresponding to the selected downlink frequencies in the Netherlands at the moment of the measurements were: 715 MHz; 847 MHz; 895 MHz; 1950 MHz and 2530 MHz.

### 2.4. Green Village Setup

**Figure 3:**
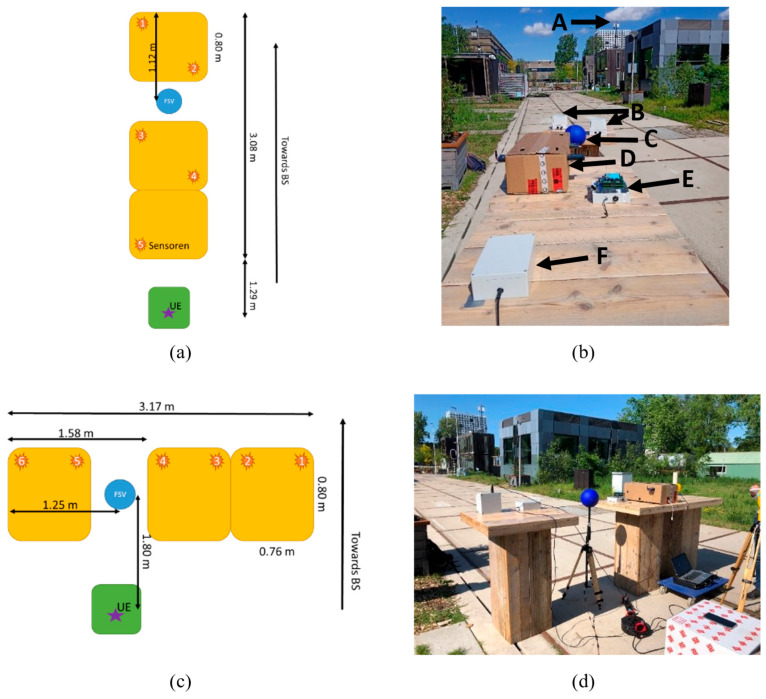
Overview of the in-situ Green Village setup. (**a**) gives an overview of the vertical configuration, (**b**) shows the real-life setup of (**a**). (**c**) shows an overview of the horizontal configuration, (**d**) shows the real-life setup (**c**). A: Base station antenna; B: WAVES sensors; C: FSV; D: SDRs combined in box (not included in this work due to wrong settings of SDRs); E: S³R sensor; F: SDR sensor.

The Green Village on the campus of Delft University of Technology is an outdoor field lab for sustainable innovation [14]. As such, a 5G NR base station is primarily installed to support various types of innovative projects but may also be used for exposure assessment experiments. This study compares two scenarios. Firstly, the sensors are placed in line with the base station (Figure 3a,b) and are sequentially repositioned to have one measurement per position (five positions in total). A “ground truth” or baseline measurement was performed with the FSV-30 setup. However, it must be noted that the FSV-30 remained fixed for the duration of the tests.

The sensors were placed on wooden tables, which have a limited reflection coefficient [15]. The tables are rectangular with sides of 0.76 m × 0.80 m and a height of 1.1 m. The sensors were placed on a diagonal of the table leaving a 0.1 m distance between the edges of the table and the sensor. The FSV-30 is placed in between the tables and is fixed at the same height as the sensors. The distance from the BS to the antenna of the FSV-30 is 49.1 m. The UE is placed on a box that is placed 1.3 m behind the last table at a height of 0.7 m. This setup aimed to be in the main beam of the BS and retained a straight line from UE to FSV to BS.

For the second scenario, the setup was rotated 90 degrees (Figure 3c,d), meaning that the sensors are now moving horizontally in the main beam. Again, the FSV-30 setup remained stationary; however, the SRM-3006 was also shifted with the sensors to verify the influence of the displacement with respect to the line base station-FSV30-UE. The SRM-3006 was rotated only once, as the goal was to investigate the influence of the variation of the sensors due to the different positions. The SRM worked in spectrum mode, with a bandwidth of 100 MHz and an RBW of 1 MHz. The measurement range was set to 3.2 V/m. The same wooden tables were used, but now the sensors were all placed along a line as can be seen in Figure 3c,d. The distance between the UE and FSV was 1.8 m while the distance between the FSV and the BS was 49.0 m.

The signal of the base station had a bandwidth of 100 MHz with a center frequency of 3.65 GHz. The base station was a standalone 5G base station and had a Time Division Duplex (TDD) implementation. The control signals (signal synchronization block (SSB)) were sent each 20 ms.

## 3. Results and Discussions

### 3.1. GTEM Cell

#### 3.1.1. Downlink Power Sweep

**Figure 4:**
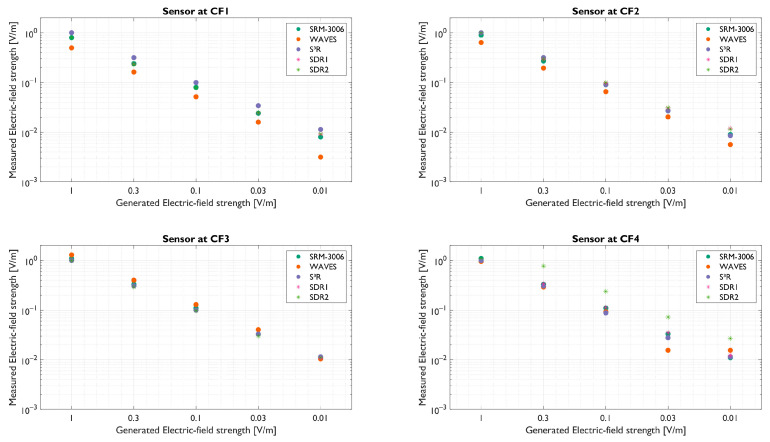
GTEM cell testing for four frequencies. The SRM-3006 is used as baseline. CF1: 773 (S³R and SDR), 806 MHz (WAVES); CF2: 940 MHz (WAVES), 1472 MHz (S³R and SDR); CF3: 1840 MHz (WAVES), 2140 MHz (S³R and SDR); CF4: 3600 MHz (WAVES, S³R and SDR).

In the GTEM cell, the linearity and sensitivity were investigated for the three RF-EMF sensors. The sensitivity is defined as the lowest field value that can be measured. The linearity of the sensor equals the log slope, which should be in accordance with the input. The accuracy, i.e., the difference in absolute value between the input and the output, could be considered as well. However, as the data gathered in the GTEM cell are used to calibrate the sensors, the accuracy will be biased and thus not discussed. Nevertheless, accurate calibration is needed before the sensors can be deployed. Figure 4 shows the result of the downlink power sweep. The sensors are grouped per center frequency (CF), from the lowest to the highest and can be found in Section 2.1. The center frequencies used for the SDRs are equal to the S³R group sensor as this study was performed in the Netherlands and these are covering the telecom bands present in the Netherlands.

For all the sensors, the linearity is clearly seen for all frequencies (i.e., log slope should equal −1). However, the measured linearity is not perfect (i.e., log slope is not equal to −1). The worst-case slope for the WAVES sensor was −1.08 at CF4, meaning that the sensor response gradually declined with respect to the ideal slope. For the S³R sensor, the worst-case slope was −1.04 at CF2. The worst-case slope of SDR1 increased with regard to the ideal slope, with a worst case slope of −0.96 at CF2, meaning that the SDRs response gradually increased with respect to the ideal slope. SDR2 had a worst-case slope of −0.85 at CF4. The WAVES sensor could not accurately measure the lowest electric field at CF4, having reached its sensitivity level at this frequency (i.e., 0.03 V/m). This is due to the component and filter choice for the hardware sensor. The other sensors did not have this limitation.

#### 3.1.2. Out-of-Band Rejection

The two hardware sensors (WAVES and S³R) are expected to filter unwanted uplink or downlink frequencies. However, as off-the-shelf filters and low-cost PCBs are used, crosstalk is expected. The measured signal can thus be a combination of the desired frequency and crosstalk. For the WAVES sensor, the frequencies, CF1, CF2 and CF3, the power reduction was 37.34 dB, 21.78 dB, and 37.80 dB, respectively. The filter used for CF4 showed the worst-case response with 10.73 dB power reduction for the tested uplink and downlink frequencies (i.e., worst case over all the frequencies). For the S³R sensor, the power reduction for crosstalk was also lowest in CF4, with 6.14 dB. For the other frequencies, CF1, CF2 and CF3, the power reduction was 12.84 dB, 27.38 dB, and 11.95 dB, respectively.

However, the SDRs are not limited to assessing only four frequencies. For the SDRs, the out-of-band response was obtained by taking advantage of the 20 MHz bandwidth. The GTEM cell sent a 10 MHz bandwidth signal, meaning that the out-of-band response is determined by the 5 MHz overshoot. This worst-case maximum power reduction for crosstalk was 14.99 dB for CF2. For the other frequencies, CF1, CF3 and CF4, the power reduction was 27.69 dB, 35.55 dB, and 22.11 dB, respectively.

### 3.2. Green Village

**Figure 5:**
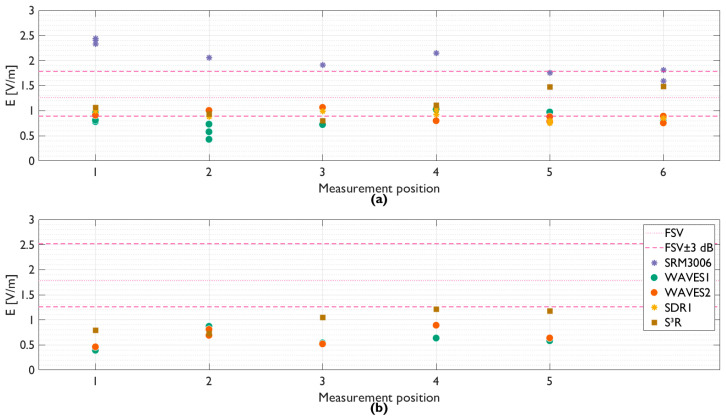
Result of in-situ testing at green village in Delft. Hardware sensors and SDR were compared to the baseline (i.e., FSV). The measurement positions (1 to 5/6) correlate with the positions indicated in Figure 3. (**a**) Horizontal configuration of Figure 3c,d; (**b**) Vertical configuration of Figure 3a,b. Red line: FSV (±3 dB is also shown because of uncertainty of SA setup [11]).

Contrary to the GTEM testing, only 5G exposure at 3.6 GHz was considered in situ to ensure that the DUT can accurately measure the new technology. Figure 5 shows the result of these measurements. Figure 5a corresponds to the situation as shown in Figure 3c,d. Figure 5b shows the situation as shown in Figure 3a,b. The red line provides the reference; measured with a setup with a Rohde & Schwarz FSV spectrum analyzer (R&S FSV SA) and measurement probe as “standard”. As there was little to no variation during the testing, this is a horizontal line. Additionally, the uncertainty interval of ± 3 dB typically linked to RF-EMF measurement setups is shown by the red dashed lines.

The devices measured EMFs between 0.09 V/m and 2.44 V/m. This is a maximum 4% of the ICNIRP limit for the electric field strength [16].

In Figure 5a, the commercially available SRM 3006 yields higher field values than the FSV, SDR, and hardware sensors (9.28 dB on average with respect to the FSV). However, both the FSV and SRM are tri-axial measurement systems while the DUT has only one vertical polarized antenna per frequency band. The difference in measured field values could be explained by the suboptimal placement of the SRM as it was placed in proximity to the DUT, which might lead to additional reflections. In addition, as the distance to the FSV increased, the measured field value was higher for the SRM and the sensors (except for the S³R sensor). It is possible that the measurement setup was slightly outside the main beam and thus that the FSV measured a lower EMF with respect to the SRM.

The sensors were placed at various measurement positions within a small area and multiple sensors of the same type (WAVES1 and WAVES2) were investigated. For WAVES1, the maximum deviation between the measurements was 2.35 dB in Figure 5a. For WAVES2, SDR and S³R, the maximum deviation was 1.38 dB, 2.05 dB, and 2.85 dB, respectively. Hence, it can be concluded that the shifting over the five and six positions over the table of the sensors had a minor influence on the EMF.

The worst-case deviation between the WAVES1 and WAVES2 sensors in Figure 5a on the same measurement position was 3.36 dB (average of 1.80 dB). The deviation could be due to differences in the manual placing of the sensors and the orientation of the antennas as small variations are possible with regard to the perfect vertical orientation.

The total average deviation (Figure 5a) between the SDR, WAVES sensor and S³R sensor was 1.86 dB, with a maximum deviation of 5.31 dB. The average and maximum deviation per measurement position is shown in Table 1. For this comparison, a combination without repetition of all sensors was used. The S^3^R sensor caused the higher deviation at Position 5 and Position 6 as can also be observed in Figure 5. It can be concluded that the sensors measured a comparable EMF.

Due to the comparison of a single-axis sensor with respect to the baseline (FSV, tri-axial), it was expected that the field values measured are lower than the baseline. However, the WAVES sensor has been rotated while remaining in the same measurement position (#2 for WAVES1 and #5 for WAVES2) to obtain a tri-axial measurement. Compared to the FSV baseline, there is then only a 1.77 dB difference, as opposed to the 9.33 dB difference between the single and tri-axial measurements (worst case).

In the second situation, Figure 5b, the SRM and SDR1 were not included in the measurements. SDR1 was rotated during the measurements (as indicated in Figure 3), but the sensor was not able to measure correctly due to incorrect settings. There was a 6.08 dB maximum deviation between the hardware sensors. The average deviation per position between the hardware sensors was 1.43 dB.

There was an average underestimation of the EMF radiation of 8.01 dB with respect to the FSV. This underestimation was expected as a tri-axial system is compared to a single-axis system.

All the tests above had an active UE present in the sector, “attracting” the beam toward the measurement setup. In the case no UE was present, the base station was sending only control signals (e.g., Synchronization Signal Block (SSB)). The FSV obtained an average field value of 0.09 V/m when there is no active UE present in the beam in both scenarios (Figure 5a,b). Both WAVES1 and WAVES2 measured an average field value over all positions and scenarios (Figure 5a,b) of 0.09 V/m. The S³R sensor measured comparable field values when an active UE was present. This is caused by the design of the sensor, a LOG filter followed by a low-pass filter, resulting in a peak detector. This design is not usable when there is no traffic or power control on the base station. The SDR measured an average EMF value of 0.10 V/m, resulting in a usable sensor to measure the control signals.

### 3.3. Discussion on the Sensors

First, the used power detector is discussed for the hardware sensors. S³R used a LOG power detector, while the WAVES sensor and SDR sensor used an RMS power detector. However, due to the fact that the LOG power detector was followed by a low-pass filter, a peak detector was created in the S³R sensor. Hence, the control signal was coupled into the signal at all times, leading to a high, unrealistic field prediction in the case that no UE was actively attracting the beam towards the measurement setup. The RMS power detector could clearly distinguish the periods where no UE was actively used, as discussed in Section 3.2. It is thus recommended that a (true) RMS detector filter is used in these sensors. A LOG filter can also be used, although not in combination with a low-pass filter.

Second, the used filters for the considered 3.5 GHz band can be improved to ensure higher power reduction for an out-of-band signal as the current power reduction is 6 dB or 11 dB for the S³R and WAVES sensors, respectively. This can be conducted either by designing a microstrip filter, cascading several filters or finding a commercially available filter that has a better response than the currently used filter.

Third, a discussion about the advantages and disadvantages of the SDR (Adalm Pluto) versus the hardware sensors (S³R, WAVES) is made. The hardware sensors allow for easy plug-and-play deployment for an extended period of time. It is possible to change the method in which the data are sent to a backend without much effort (e.g., WiFi, LoRa, ethernet). Thanks to the use of off-the-shelf components, a low-cost sensor (<1000 euro) is created, allowing for multiple sensors even with a limited budget. The purpose of these sensors is to monitor the time behavior of the investigated telecom and 5G NR signals.

However, they can measure only one vector component of a number of fixed frequency bands (here, four bands are measured). This can be extended to measure more frequency bands and other vector components can be added as well. To measure a different frequency band, it is likely that a new PCB layout is needed, hindering a fast rollout of new measurement sensors. The considered hardware sensors are connected to the main power grid, making the hardware sensors less flexible to install. However, these hardware sensors can also be powered by batteries, extending their flexibility.

The SDR on the other hand can be programmed to sequentially sample all frequency bands of interest, leading to a highly flexible measurement system. Hence, this results in a frequency-selective measurement system. Demodulation and decoding of the signal are possible if the correct sampling rate can be reached. Despite that, this leads to a lower sampling speed than the hardware sensors (i.e., the SDR can focus on only one band at the time). The SDR also leads to a huge amount of data, needing higher computation power to process the measurements.

Long-term evolutions of these low-cost sensors must be quantified as was conducted in [6] and extended with the amount of drift, and the influence of weather conditions. Lastly, more research is needed on the initial placement, more specifically on the environmental conditions, e.g., the influence of placing a sensor close to a wall, and the influence of sensor height needs to be considered.

## 4. Conclusions and Future Work

This study compared low-cost hardware sensors and SDR sensors with expensive verified measurement setups consisting of spectrum analyzer equipment for RF-EMF radiation. Both in-lab (GTEM cell) and in-situ measurements have been performed for this comparison. The in-lab testing showed the importance of selecting the correct components for the hardware sensors to ensure shielding from crosstalk. Furthermore, an in-lab calibration must be performed to guarantee an accurate response to real-life exposure.

The in-situ testing confirmed that the low-cost hardware sensors and SDR can be used to assess the RF-EMF radiation. Values between 0.09 V/m and 2.44 V/m were obtained at a distance of about 50 m from the base station. The variability between the sensors was 1.78 dB on average, with a maximum deviation of 5.26 dB. However, it must be kept in mind that these RF-EMF sensors only measured one vector component (purpose of temporal monitoring) of the field, and therefore, the given field will be an underestimation of the total field at that measurement location.

These devices can be used to provide the general public and governments only temporal and spatial 5G field behavior values. These data can then be combined with more accurate measurement systems to create highly accurate spatial-temporal EMF radiation maps.

Future research on these sensors entails including the maximum measurable field level to verify the extendibility of the conclusions presented in this paper in case of high EMFs. In addition, new tri-axial sensors must be constructed which can measure all three vector components. In addition, mm-waves hardware sensors must be made to cover all 5G NR frequencies (FR2). Furthermore, the current sensors could be recalibrated so that their response is mapped to tri-axial measurement devices.

## Figures and Tables

**Table 1:** Overview of the average and maximum deviation between the SDR, WAVES1, WAVES2 and S^3^R sensor.

	Position 1	Position 2	Position 3	Position 4	Position 5	Position 6
Average Deviation (dB)	0.74	1.46	1.99	1.44	2.80	2.70
Maximum Deviation (dB)	1.36	2.76	3.37	2.83	5.31	5.26

## Data Availability

The datasets generated during and/or analyzed during the current study are available from the corresponding author upon reasonable request.
